# The Northern Ohio Dental Association

**Published:** 1871-06

**Authors:** 


### THE NORTHERN OHIO DENTAL ASSOCIATION.

The Northern Ohio Dental Association met at Put-in-Bay, on Tuesday, May 2nd, with quite a large number of the members in attendance. The sessions continued two days, and were interesting indeed. The discussions were upon important practical subjects, and were participated in by nearly all present. New thoughts and ideas were received by all present.

The location, doubtless, had much to do in making so good a meeting, it is one most charming—one of the most lovely spots anywhere to be found.

Put-in-Bay, with its matchless beauties, its splendid scenery, its ample hotels with their fine appointments, its genial West and Sweney, all together combine to make this one of the most pleasant and delightful places anywhere to be found.

So well pleased were all the members of the society, that it was unanimously voted to hold the next annual meeting at the same place; indeed, they thought it totally impracticable to try to hold it anywhere else, for certainly all who went there will go next year, and endeavor to induce every body else to go. T.

